# Co‐Infection of Mucormycosis and COVID‐19: A Retrospective Cross‐Sectional Study of Patients Admitted to Imam Khomeini Hospital in Ahvaz, Iran

**DOI:** 10.1002/hsr2.70831

**Published:** 2025-05-19

**Authors:** Javad Zarei, Hanieh Raji, Seyed Mohammad Alavi, Farid Yousefi, Nasrin Amirrajab

**Affiliations:** ^1^ Department of Health Information Technology, School of Allied Medical Sciences Ahvaz‎ Jundishapur University of Medical Sciences Ahvaz Iran; ^2^ Department of Internal Medicine, Air Pollution and Respiratory Diseases Research Center Ahvaz Jundishapur University of Medical Sciences Ahvaz Iran; ^3^ Infectious and Tropical Diseases Research Center, Health Research Institute Ahvaz Jundishapur University of Medical Sciences Ahvaz Iran; ^4^ Health Research Institute, Infectious and Tropical Diseases Research Center Ahvaz Jundishapur University of Medical Sciences Ahvaz Iran; ^5^ Department of Laboratory Sciences, School of Allied Medical Sciences Ahvaz Jundishapur University of Medical Sciences Ahvaz Iran; ^6^ Nutrition and Metabolic Disease Research Center, Clinical Sciences Research Institute Ahvaz Jundishapur University of Medical Sciences Ahvaz Iran

**Keywords:** co‐infection, COVID‐19 virus disease, fungal infections, Mucormycosis

## Abstract

**Background and Aims:**

During the COVID‐19 pandemic, the emergence of mucormycosis, a rare but often fatal fungal infection, gained significant attention due to its increased prevalence among immunocompromised patients. This study aimed to determine the prevalence and characterize the clinical features of COVID‐19‐associated mucormycosis in patients admitted to Imam Khomeini Hospital, Ahvaz, Iran.

**Methods:**

This retrospective, cross‐sectional study analyzed clinical data from patients admitted between November 2020 and November 2021. Inclusion criteria encompassed individuals with concurrent diagnoses of COVID‐19 and mucormycosis. Data collected included demographic details, clinical features, comorbidities, laboratory findings, and treatment information. Descriptive statistics were used to summarize patient characteristics, and prevalence estimates were provided with 95% confidence intervals.

**Results:**

Of the 12,978 hospitalized patients during the study period, 31 individuals (0.2%, 95% CI: 0.2%–0.3%) were diagnosed with COVID‐19‐associated mucormycosis. The prevalence was highest among male patients (54.8%) and those aged 60 years or older (48.4%). Diabetes was the most prevalent comorbidity, and the most frequent clinical symptoms included respiratory distress and cough. Patients with severe COVID‐19 exhibited a significantly higher prevalence of co‐infection, suggesting a greater vulnerability in this subgroup.

**Conclusion:**

COVID‐19‐associated mucormycosis primarily affects elderly male patients with underlying diabetes and severe COVID‐19 illness. These findings underscore the importance of early detection and intervention, particularly for high‐risk individuals. Further research is warranted to optimize prevention and management strategies for this serious complication.

## 1 Introduction

The Coronavirus disease 2019 (COVID‐19) pandemic, driven by the severe acute respiratory syndrome coronavirus 2 (SARS‐CoV‐2), has placed immense pressure on global healthcare systems, leading to millions of cases and fatalities worldwide. COVID‐19 presents with a broad clinical spectrum, ranging from mild respiratory symptoms to severe and potentially fatal complications [1]. Hospitalized COVID‐19 patients, especially those with underlying health conditions or in intensive care units (ICUs), are at high risk of secondary infections due to their immunocompromised status, prolonged hospital stays, and extensive use of invasive devices such as ventilators [2, 3, 4].

Mucormycosis, commonly called “black fungus”, is a highly aggressive fungal infection that predominantly affects immunocompromised individuals, such as those with uncontrolled diabetes or patients on immunosuppressive therapies. This infection is characterized by its rapid progression, often involving the sinuses, brain, and lungs, and can lead to high mortality rates if not detected and treated early [4, 5]. The concurrence of COVID‐19 and mucormycosis has created a complex and high‐risk co‐infection scenario, especially in patients subjected to prolonged ICU stays, high‐dose corticosteroid therapy, and severe hyperglycemia, which collectively increase mortality risks [5, 6].

In 2021, several countries, particularly India, reported a surge in mucormycosis cases among patients recovering from COVID‐19, highlighting an urgent need to understand this coinfection's prevalence, associated risk factors, and clinical outcomes [4, 7, 8, 9, 10, 11, 12]. However, limited data are available on COVID‐19‐associated mucormycosis in other regions, including the Middle East. Therefore, this study aims to examine the epidemiological and clinical characteristics of COVID‐19‐associated mucormycosis in patients admitted to Imam Khomeini Hospital in Ahvaz, Iran. Understanding these features can better inform clinical management practices and public health strategies to address this emerging healthcare challenge.

## 2 Materials and Methods

### 2.1 Study Design and Participants

This retrospective, cross‐sectional study analyzed clinical records of patients admitted to Imam Khomeini Hospital in Ahvaz, Iran, between November 2020 and November 2021. Ethical approval was obtained from the Ahvaz Jundishapur University of Medical Sciences (IR.AJUMS.REC.1400.484), and the study adhered to the Helsinki Declaration. The study population included patients diagnosed with both COVID‐19 and mucormycosis. The study population included patients diagnosed with both COVID‐19 and mucormycosis. COVID‐19 was confirmed through a positive RT‐PCR test for SARS‐CoV‐2. At the same time, mucormycosis diagnosis was based on clinical, radiological, and histopathological assessments following criteria established by the European Organization for Research and Treatment of Cancer/Invasive Fungal Infections Cooperative Group and the Mycoses Study Group Education and Research Consortium (EORTC/MSGERC) [13, 14, 15].

### 2.2 Patient Demographics and Comorbidities

The retrospective study primarily consisted of patients aged 60 years and older, a demographic more susceptible to COVID‐19‐associated mucormycosis. Key comorbidities observed included diabetes and hypertension, both of which are known risk factors for mucormycosis. Additionally, some of the patients had a history of heart disease, although conditions such as immunodeficiency and chronic lung disease were not present in this study.

### 2.3 Data Collection

**Figure 1:**
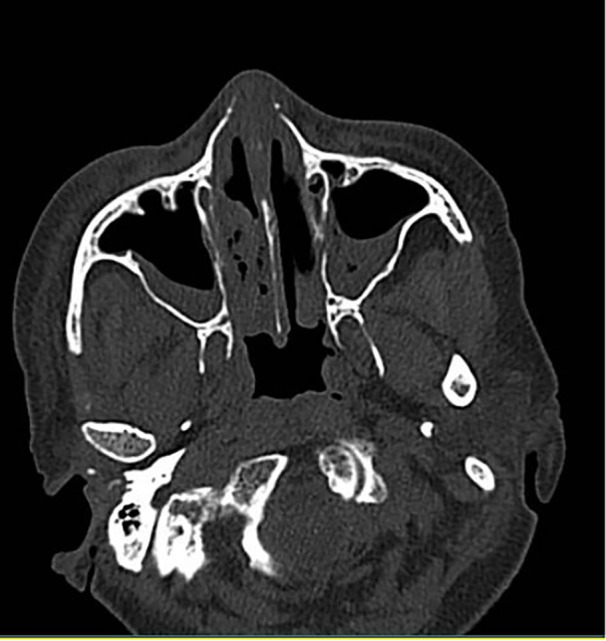
Axial CT scan of a patient with COVID‐19‐associated mucormycosis. This non‐enhanced axial CT scan shows notable mucosal thickening in the bilateral maxillary sinuses, consistent with sinonasal mucormycosis. The image also reveals partial opacification of both maxillary sinuses and the right nasal cavity, accompanied by air entrapment. These findings are characteristic of mucormycosis and illustrate the extent of sinonasal involvement in a patient concurrently diagnosed with COVID‐19.

**Figure 2:**
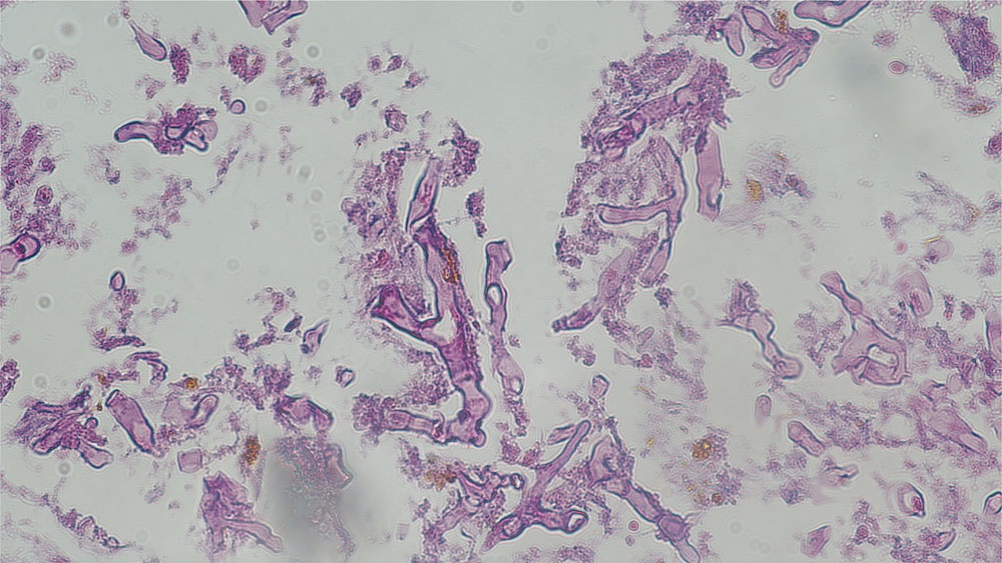
Histopathological analysis of nasal mucosal glands in COVID‐19‐associated Mucormycosis. This histopathological smear from the left maxillary sinus highlights nasal mucosal glands with evident necrosis and mononuclear inflammatory cell infiltration, characteristic of mucormycosis. Broad, nonseptate, or pauciseptate hyphae are visible, displaying wide‐angle branching and significant tissue invasion. These fungal structures, typical of mucormycosis, are stained with Hematoxylin and Eosin at a magnification of 200×.

Demographic and clinical data, including age, gender, COVID‐19 severity, comorbid conditions, and in‐hospital outcomes, were extracted from patient medical records. Laboratory parameters, such as blood glucose levels, C‐reactive protein (CRP), and creatinine, were documented alongside clinical features, including corticosteroid use, antibiotic administration, and prior surgical history. Radiological evaluations, such as CT scans of the orbit, paranasal sinuses, and lungs, were performed (Figure 1), and nasal mucosal biopsies underwent histopathological staining with hematoxylin–eosin (H&E) and Gomori methenamine silver (GMS) to confirm mucormycosis (Figure 2).

### 2.4 Sample Size and Data Analysis

To estimate the frequency of co‐infection, all patients meeting the inclusion criteria during the specified timeframe were retrospectively enrolled in this study. Thoroughly trained medical professionals reviewed each patient's medical records, ensuring data accuracy and consistency. These records were then anonymized for confidentiality. Data were analyzed using STATA version 14, and a 95% confidence interval (CI) was calculated to assess the association between co‐infection and demographic factors, comorbidities, and COVID‐19 severity.

## 3 Results

**Figure 3:**
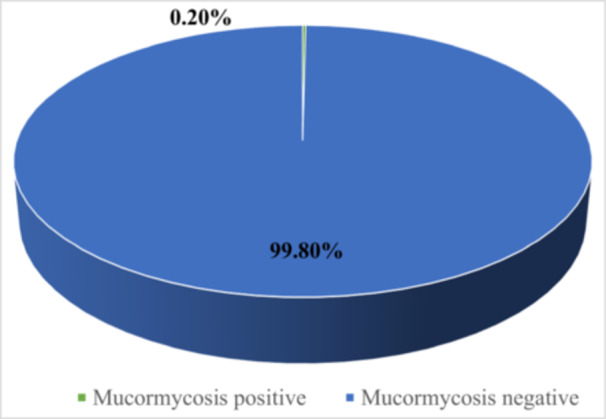
Frequency of Mucormycosis infection among assessed COVID‐19 patients.

Among the 12,978 hospitalized patients during the study period, 31 individuals (0.2%, 95% CI: 0.2%–0.3%) were identified with COVID‐19‐associated mucormycosis. The prevalence rate was highest in male patients and those aged 60 years or older. Concurrent infections were more frequent in patients with severe cases of COVID‐19. The most common symptoms observed were respiratory distress, cough, and fever, while diabetes was the most prevalent comorbidity (Figure 3).

### 3.1 Demographic Differentiation

The highest prevalence of mucormycosis related to COVID‐19 was observed in patients aged 60 years or older, with 48.4% presenting with co‐infections, compared to only 16.1% of those younger than 40. Among male patients, 54.8% had concurrent infections, whereas 45.2% were female (Table 1).

**Table 1:** Prevalence of mucormycosis infection associated with COVID‐19 based on demographic differentiation.

Characteristics		Number	%^a^	95% CI low^b^	95% CI up^b^
Age group (years)	40 > years	5	16.1	6.5	34.6
	40–59 years	11	35.5	20.2	54.5
	≥ 60 years	15	48.4	30.8	66.4
Gender	Male	17	54.8	36.5	72.0
	Female	14	45.2	28.0	63.5

^a^ Percentage of total patients with mucormycosis who were also diagnosed with COVID‐19.

^b^ 95% CI (Confidence Interval) up and 95% CI low indicate the upper and lower limits of the 95% confidence interval, respectively.

### 3.2 COVID‐19 Severity

The prevalence of concurrent mucormycosis was 61.3% (95% CI: 43.1%–79.5%) in patients with severe COVID‐19. In moderately ill patients, the prevalence was 6.5% (95% CI: −2.7%–15.6%); in patients with mild cases, it was 32.2% (95% CI: 14.8%–49.7%) (Table 2).

**Table 2:** The prevalence of COVID‐19‐associated mucormycosis‐based COVID‐19 severity.

COVID‐19 severity	Number	%^a^	95% CI low^b^	95% CI up^b^
Severe	19	61.3	43.1	79.5
Moderate	2	6.5	−2.7	15.6
Mild	10	32.2	14.8	49.7

^a^ Percentage of total patients with mucormycosis who were also diagnosed with COVID‐19.

^b^ 95% CI (Confidence Interval) up and 95% CI low indicate the upper and lower limits of the 95% confidence interval, respectively.

### 3.3 Clinical Symptoms

The most prevalent symptoms in coinfected patients were respiratory distress (67.7%), cough (58.1%), and fever (19.4%). Less common symptoms included headache (13.0%) and loss of taste (6.5%). None of the coinfected cases displayed symptoms such as limb plegia, nausea, loss of smell, seizures, abdominal pain, or diarrhea (Table 3).

**Table 3:** The prevalence of COVID‐19‐associated mucormycosis based on clinical symptom differentiation.

Clinical symptoms	Number	%^a^	95% CI low^b^	95% CI up^b^
Fever	6	19.4	8.5	38.2
Cough	18	58.1	39.4	74.7
Chest pain	1	3.2	0.4	21.6
Muscular pain	5	16.1	6.2	34.6
Respiratory distress	21	67.7	48.6	82.3
Loss of consciousness	5	16.1	6.5	34.6
Loss or reduction of the sense of smell	0	0	N/A	N/A
Losing or reducing the sense of taste	2	6.5	1.5	23.9
Seizure	0	0	N/A	N/A
Abdominal pain	0	0	N/A	N/A
Headache	4	13.0	4.6	31.1
Nausea	0	0	—	38.2
Vomit	1	3.2	0.4	21.6
Anorexia	2	6.5	1.5	23.9
Dizziness	2	6.5	1.5	23.9
Muscular pain	5	16.1	6.2	34.6
Diarrhea	0	0	N/A	N/A
Limb Plegia	0	0	N/A	N/A

Abbreviation: N/A, not applicable.

^a^ Percentage of total patients with mucormycosis who were also diagnosed with COVID‐19.

^b^ 95% CI (Confidence Interval) up and 95% CI low indicate the upper and lower limits of the 95% confidence interval, respectively.

### 3.4 Risk Factors

Diabetes was the most frequent risk factor, observed in 19.4% of coinfected patients, followed by hypertension (13.0%) and heart disease (6.5%). Mortality among coinfected cases was 80.7%, underscoring the severity of this co‐infection (Table 4).

**Table 4:** Prevalence of COVID‐19‐associated mucormycosis by considering various risk factors.

Risk factor	Number	%^a^	95% CI low^b^	95% CI up^b^
Diabetes	6	19.4	8.5	38.2
Chronic blood diseases	0	0	—	—
Heart disease	2	6.5	1.5	23.9
Smoking	0	0	—	—
Drug abuse	0	0	—	—
Cancer	0	0	—	—
Chronic liver diseases	0	0	—	—
Pregnant	0	0	—	—
Immunodeficiency	0	0	—	—
HIV/AIDS	0	0	—	—
Inflammation	0	0	—	—
Hypertension	4	13.0	4.7	31.1
Chronic kidney diseases	1	3.2	0.4	21.6
Asthma	0	0	—	—
Other chronic lung diseases, except asthma	0	0	—	—
Chronic neurological disorders	0	0	—	—
Mortality	25	80.7	61.9	91.5

^a^ Percentage of total patients with mucormycosis who were also diagnosed with COVID‐19.

^b^ 95% CI (confidence interval) up and 95% CI low indicate the upper and lower limits of the 95% confidence interval, respectively.

### 3.5 Detection Factors

Among the diagnostic and clinical factors, 38.7% of coinfected patients had hypoxia (PO_2_ < 93), while 61.3% did not. All cases were confirmed by CT imaging and histopathology. Elevated blood glucose (> 115 mg/dL) was present in 86.2% of cases, and most had elevated CRP levels (84.6%), indicating significant inflammation. Corticosteroid exposure was documented in 45.2% of cases, antibiotic use in 80.6%, and prior surgeries in 38.7% (Table 5).

**Table 5:** Prevalence of COVID‐19‐associated mucormycosis based on detection factors.

Detection Factor		Number	%^a^	95% CI low^b^	95% CI up^b^
Level PO_2_	< 93	12	38.7	22.7	57.6
	> 93	19	61.3	42.4	77.3
CT scan	Positive	29	96.7	77.8	99.6
	Negative	1	3.3	0.4	22.2
RT‐PCR	Positive	31	100.0	—	—
	Negative	0	0.0	—	—
Histopathology	Positive	31	100.0	—	—
	Negative	0	0.0	—	—
Creatinine	Low (< 6.0 mg/dL)	6	19.3	8.8	38.1
	Medium (6.0–20.0 mg/dL)	21	67.7	48.6	82.3
	High (> 20.0 mg/dL)	4	13.0	4.7	31.1
Glucose	Low (< 75 mg/dL)	0	0.0	—	—
	Medium (75–115 mg/dL)	4	13.8	4.9	33.0
	High (> 115 mg/dL)	25	86.2	67.0	95.1
CRP §	Positive	22	84.6	63.5	94.5
	Negative	4	15.4	5.5	36.3
Cortone use	Yes	14	45.2	28.0	63.5
	No	17	54.8	36.5	72.0
Antibiotic use	Yes	25	80.6	61.9	91.5
	No	6	19.4	8.5	38.1
Surgery	Yes	12	38.7	22.7	57.6
	No	19	61.3	42.4	77.3

Abbreviation: CRP §, C‐reactive protein.

^a^ Percentage of total patients with mucormycosis who were also diagnosed with COVID‐19.

^b^ 95% CI (Confidence Interval) up and 95% CI low indicate the upper and lower limits of the 95% confidence interval, respectively.

## 4 Discussion

This retrospective study identified a 0.2% prevalence of COVID‐19‐associated mucormycosis among hospitalized patients, a rate that aligns with the lower range of prevalence figures reported in studies from India, where prevalence estimates have ranged from 0.2% to 1.8% among COVID‐19 cases [7, 16, 17, 18, 19]. The relatively higher prevalence documented in some Indian studies has been attributed to various factors, including high corticosteroid usage in COVID‐19 treatment protocols, environmental exposure to fungal spores, and the substantial prevalence of uncontrolled diabetes, which is a significant risk factor for mucormycosis [4, 6].

The comparatively lower prevalence observed in this study may reflect regional differences in corticosteroid administration practices, variations in healthcare protocols, and SARS‐CoV‐2 strain diversity. Certain aggressive strains of the virus have been associated with an increased incidence of mucormycosis in some studies, suggesting that regional strain variability may influence co‐infection prevalence [7, 20]. Furthermore, the differences in mucormycosis prevalence across geographic areas underscore the impact of local environmental and clinical factors, such as fungal spore exposure and ICU care protocols, on infection rates [10, 11]. In Middle Eastern regions, where environmental and clinical conditions differ from those in South Asia, a lower prevalence of mucormycosis may be expected, which is in line with our study findings.

Emerging studies from other regions, including Southeast Asia and the United States, have reported prevalence rates lower than those documented in India, supporting the hypothesis that local clinical practices, including controlled corticosteroid use and proactive diabetes management, may play a critical role in reducing mucormycosis risk [21, 22]. These findings highlight the importance of region‐specific clinical guidelines and infection control practices in managing COVID‐19 patients and preventing secondary fungal infections such as mucormycosis.

Despite the relatively low prevalence, concurrent mucormycosis represents a serious risk among severe COVID‐19 cases, particularly for immunocompromised patients, elderly individuals, and those requiring extended ICU admission [4, 20, 23]. Patients aged 60 years and older in our study exhibited a markedly higher rate of co‐infection, likely due to age‐associated immunosenescence and the prevalence of comorbidities, which have been shown to increase susceptibility to opportunistic fungal infections [24, 25, 26]. This pattern underscores the importance of vigilant monitoring for fungal superinfections in older COVID‐19 patients, especially those with other risk factors.

Our findings underscore the prominent role of diabetes as a major risk factor, observed in 19.4% of COVID‐19‐associated mucormycosis cases in this study. This aligns with previous reports indicating diabetes prevalence between 15% and 70% in coinfected patients, highlighting the critical role of hyperglycemia in fostering fungal growth while compromising immune responses to Mucorales fungi [6, 27]. Additionally, the observed prevalence of hypertension (13%) is consistent with other studies and suggests that hypertension may compound COVID‐19's pro‐thrombotic and inflammatory effects, increasing vulnerability to mucormycosis [28, 29].

Furthermore, 38.7% of mucormycosis patients in our study presented with hypoxia, which aligns with previous reports indicating high rates of respiratory distress in coinfected patients [21, 22, 30]. Hypoxia and other respiratory symptoms, such as dyspnea and cough, are common among mucormycosis patients and may exacerbate respiratory complications in COVID‐19, further impairing oxygenation and leading to poorer outcomes. Co‐infection among non‐hypoxic patients in our study underscores the critical need for broader screening practices for mucormycosis in COVID‐19 patients, regardless of respiratory symptoms. This is particularly relevant as latent or subclinical mucormycosis infections may progress undetected, leading to severe complications. Cornely et al. and Samson and Dharne emphasize that early identification of asymptomatic or mildly symptomatic cases can prevent the escalation of invasive fungal disease [31, 32]. Moreover, Dilek et al. [22] suggest that screening protocols inclusive of non‐hypoxic COVID‐19 patients could improve early detection and treatment outcomes by identifying fungal colonization before it advances to more severe disease states.

Furthermore, 38.7% of mucormycosis patients in our study presented with hypoxia, which aligns with previous reports indicating high rates of respiratory distress in coinfected patients [21, 22, 30]. However, the presence of co‐infection in non‐hypoxic patients highlights the necessity of screening for mucormycosis across all COVID‐19 patients, regardless of respiratory symptoms, to identify potential latent fungal infections.

Diagnostic confirmation through universal RT‐PCR and CT positivity in this study supports the reliability of these tools for detecting COVID‐19 in patients with mucormycosis, even in cases without typical hypoxic symptoms [31, 33]. The frequent observations of glucose dysregulation and heightened inflammatory markers in coinfected cases further reinforce the interplay between hyperinflammation, hyperglycemia, and mucormycosis risk in COVID‐19 patients [4]. Although corticosteroid use was documented in nearly half of the cases, careful glucocorticoid management remains essential to mitigate infection risk without exacerbating hyperglycemic conditions.

Diagnostic confirmation of Mucor infection in this study was based on clinical, radiological, and histopathological criteria, which are widely accepted for the diagnosis of mucormycosis. As highlighted in recent literature, RT‐PCR analysis of Mucorales in tissue or biopsy samples can detect probable mucormycosis infection up to 11 days before standard diagnostic methods, offering a significant advantage for early intervention [34, 35].

While this study provides valuable insights into the epidemiological and clinical characteristics of COVID‐19‐associated mucormycosis, several limitations must be acknowledged. First, the retrospective nature of the study design limited our ability to control for confounding variables, potentially introducing selection bias, as only patients meeting stringent inclusion criteria were analyzed. Additionally, our reliance on clinical, radiological, and histopathological diagnostic methods may have resulted in an underestimation of mucormycosis cases due to potential misclassification or undiagnosed instances within the COVID‐19 patient population. We recognize the significant value of molecular diagnostic techniques, such as RT‐PCR, for the early and accurate identification of Mucorales, and we emphasize the importance of incorporating these advanced methods in future research to enhance diagnostic precision and guide targeted therapeutic interventions. Furthermore, the absence of a control group comprising COVID‐19 patients without mucormycosis restricts our ability to draw direct comparative conclusions regarding symptom profiles and risk factors specific to mucormycosis. Lastly, due to resource constraints, molecular diagnostic techniques—which could have improved pathogen specificity—were not employed. Future studies utilizing a prospective, multicenter design, along with molecular diagnostic tools and a more comprehensive control group, are essential to validate our findings and to further elucidate the complex relationship between COVID‐19 and mucormycosis.

## 5 Conclusion

These findings align with global reports on the demographics, clinical presentations, comorbidities, and prognosis of patients with COVID‐19 and secondary mucormycosis. The prevalence underscores the need for healthcare providers to maintain high clinical vigilance for signs of opportunistic fungal infections, especially in older males with diabetes presenting with typical COVID‐19 respiratory symptoms. Early detection and management of diabetes, along with cautious corticosteroid use, are prudent in these high‐risk patients.

The high mortality rate of 80.7% among coinfected patients in this study highlights the severe and life‐threatening nature of COVID‐19‐associated mucormycosis. This alarming mortality rate underscores the urgent need for early diagnosis, aggressive treatment, and preventive measures to reduce the devastating impact of this co‐infection.

Further collaborative studies are necessary to confirm population‐based estimates, elucidate infection mechanisms, and standardize prevention and treatment protocols for this life‐threatening COVID‐19 complication. Our findings provide essential insights to guide clinical vigilance and timely interventions that could mitigate mucormycosis occurrence and its severe consequences in vulnerable hospitalized COVID‐19 patients. Future studies should aim to incorporate molecular characterization techniques to improve the specificity of mucor isolate identification and potentially reveal species‐specific clinical patterns.

## Author Contributions

**Javad Zarei:** data curation, project administration, and supervision. **Hanieh Raji:** methodology and data curation. **Seyed Mohammad Alavi:** data curation, supervision, and conceptualization. **Farid Yousefi:** methodology and data curation. **Nasrin Amirrajab:** data curation, software, writing – original draft, investigation, formal analysis, validation, writing – review and editing, and visualization.

## Conflicts of Interest

The authors declare no conflicts of interest.

## Transparency Statement

The lead author Nasrin Amirrajab affirms that this manuscript is an honest, accurate, and transparent account of the study being reported; that no important aspects of the study have been omitted; and that any discrepancies from the study as planned (and, if relevant, registered) have been explained.

## Data Availability

The data that support the findings will be made available on request from the corresponding author.

## References

[1] World Health Organization , “WHO Coronavirus (COVID‐19) Dashboard,” published 2023, https://covid19.who.int/.

[2] W. Guan , Z. Ni , Y. Hu , et al., “Clinical Characteristics of Coronavirus Disease 2019 in China,” New England Journal of Medicine 382, no. 18 (2020): 1708–1720. 32109013 10.1056/NEJMoa2002032 PMC7092819

[3] Z. Wu and J. M. McGoogan , “Characteristics of and Important Lessons From the Coronavirus Disease 2019 (COVID‐19) Outbreak in China: Summary of a Report of 72 314 Cases From the Chinese Center for Disease Control and Prevention,” Journal of the American Medical Association 323, no. 13 (2020): 1239–1242. 32091533 10.1001/jama.2020.2648

[4] A. K. Singh , R. Singh , S. R. Joshi , and A. Misra , “Mucormycosis in COVID‐19: A Systematic Review of Cases Reported Worldwide and in India,” Diabetes & Metabolic Syndrome 15, no. 4 (2021): 102146. 34192610 10.1016/j.dsx.2021.05.019 PMC8137376

[5] A. Patel , H. Kaur , I. Xess , et al., “A Multicentre Observational Study on the Epidemiology, Risk Factors, Management and Outcomes of Mucormycosis in India,” Clinical Microbiology and Infection 26, no. 7 (2020): 944.e9–944.e15. 10.1016/j.cmi.2019.11.021 31811914

[6] T. M. John , C. N. Jacob , and D. P. Kontoyiannis , “When Uncontrolled Diabetes Mellitus and Severe COVID‐19 Converge: The Perfect Storm for Mucormycosis,” Journal of Fungi 7, no. 4 (2021): 298. 33920755 10.3390/jof7040298 PMC8071133

[7] A. Patel , R. Agarwal , S. M. Rudramurthy , et al., “Multicenter Epidemiologic Study of Coronavirus Disease–Associated Mucormycosis, India,” Emerging Infectious Diseases 27, no. 9 (2021): 2349–2359. 34087089 10.3201/eid2709.210934 PMC8386807

[8] H. Prakash and A. Chakrabarti , “Global Epidemiology of Mucormycosis,” Journal of Fungi 5, no. 1 (2019): 26. 30901907 10.3390/jof5010026 PMC6462913

[9] S. Challa , “Mucormycosis: Pathogenesis and Pathology,” Current Fungal Infection Reports 13 (2019): 11–20. 10.1007/s12281-022-00443-z PMC9520103 36193101

[10] A. Skiada , C. Lass‐Floerl , N. Klimko , A. Ibrahim , E. Roilides , and G. Petrikkos , “Challenges in the Diagnosis and Treatment of Mucormycosis,” Medical Mycology 56, no. S1 (2018): S93–S101. 10.1093/mmy/myx101 PMC6251532 29538730

[11] W. Jeong , C. Keighley , R. Wolfe , et al., “The Epidemiology and Clinical Manifestations of Mucormycosis: A Systematic Review and Meta‐Analysis of Case Reports,” Clinical Microbiology and Infection 25, no. 1 (2019): 26–34. 30036666 10.1016/j.cmi.2018.07.011

[12] G. Hamilos , G. Samonis , and D. Kontoyiannis , “Pulmonary Mucormycosis,” Seminars in Respiratory and Critical Care Medicine 32, no. 06 (2011): 693–702. 22167397 10.1055/s-0031-1295717

[13] B. De Pauw , T. J. Walsh , J. P. Donnelly , et al., “Revised Definitions of Invasive Fungal Disease From The European Organization for Research and Treatment of Cancer/Invasive Fungal Infections Cooperative Group and the National Institute of Allergy and Infectious Diseases Mycoses Study Group (EORTC/MSG) Consensus Group,” Clinical Infectious Diseases 46, no. 12 (2008): 1813–1821. 18462102 10.1086/588660 PMC2671227

[14] M. Bassetti , E. Azoulay , B.‐J. Kullberg , et al., “EORTC/MSGERC Definitions of Invasive Fungal Diseases: Summary of Activities of the Intensive Care Unit Working Group,” Clinical Infectious Diseases 72, no. S2 (2021): S121–S127. 33709127 10.1093/cid/ciaa1751

[15] J. P. Donnelly , S. C. Chen , C. A. Kauffman , et al., “Revision and Update of the Consensus Definitions of Invasive Fungal Disease From the European Organization for Research and Treatment of Cancer and the Mycoses Study Group Education and Research Consortium,” Clinical Infectious Diseases 71, no. 6 (2020): 1367–1376. 31802125 10.1093/cid/ciz1008 PMC7486838

[16] L. Selarka , S. Sharma , D. Saini , et al., “Mucormycosis and COVID‐19: An Epidemic Within a Pandemic in India,” Mycoses 64, no. 10 (2021): 1253–1260. 34255907 10.1111/myc.13353 PMC8446956

[17] G. Pasquier , “COVID‐19‐Associated Mucormycosis in India: Why Such an Outbreak?,” Journal of Medical Mycology 33, no. 3 (2023): 101393. 37182234 10.1016/j.mycmed.2023.101393 PMC10168193

[18] K. T. Sethuraman , J. A. Thiruvengadam , A. Ravichandran , and S. T. Sengottaiyan , “Prevalence, Predictors, and Outcome of Pulmonary Mucormycosis in COVID‐19 Associated Rhino Orbital Mucormycosis in a Tertiary Care Center in South India,” Current Medical Mycology 9, no. 3 (2023): 33. 10.22034/cmm.2023.345154.1486 PMC10864746 38361963

[19] S. Raychaudhuri , J. Taneja , J. Sasidharan , et al., “A Critical Appraisal of Mucormycosis in COVID‐19 Patients in a Tertiary Care Centre in India,” Current Medical Mycology 9, no. 1 (2023): 1–7. 10.18502/CMM.2023.150667 PMC10590186 37867588

[20] J. L. Hernández and C. J. Buckley , Mucormycosis (StatPearls Publishing, 2023). 31335084

[21] P. K. Rudrabhatla , A. Reghukumar , and S. V. Thomas , “Mucormycosis in COVID‐19 Patients: Predisposing Factors, Prevention and Management,” Acta Neurologica Belgica 122 (2022): 273–280. 34820787 10.1007/s13760-021-01840-w PMC8612391

[22] A. Dilek , R. Ozaras , S. Ozkaya , M. Sunbul , E. I. Sen , and H. Leblebicioglu , “COVID‐19‐Associated Mucormycosis: Case Report and Systematic Review,” Travel Medicine and Infectious Disease 44 (2021): 102148. 34454090 10.1016/j.tmaid.2021.102148 PMC8387131

[23] P. Monika and M. N. Chandraprabha , “Risks of Mucormycosis in the Current Covid‐19 Pandemic: A Clinical Challenge in Both Immunocompromised and Immunocompetent Patients,” Molecular Biology Reports 49, no. 6 (2022): 4977–4988. 35107737 10.1007/s11033-022-07160-3 PMC8808276

[24] P. Arjmand , M. Bahrami , Z. E. Mohammadie , et al., “Mucormycosis in Pre‐COVID‐19 and COVID‐19 Era: A Study of Prevalence, Risk Factors and Clinical Features,” Laryngoscope Investigative Otolaryngology 7, no. 5 (2022): 1343–1350. 36249085 10.1002/lio2.899 PMC9539365

[25] R. Sharma , P. Kumar , A. Rauf , et al., “Mucormycosis in the COVID‐19 Environment: A Multifaceted Complication,” Frontiers in Cellular and Infection Microbiology 12 (2022): 937481. 35923801 10.3389/fcimb.2022.937481 PMC9339637

[26] V. Bajaj , N. Gadi , A. P. Spihlman , S. C. Wu , C. H. Choi , and V. R. Moulton , “Aging, Immunity, and COVID‐19: How Age Influences the Host Immune Response to Coronavirus Infections?,” Frontiers in Physiology 11 (2021): 571416. 33510644 10.3389/fphys.2020.571416 PMC7835928

[27] W. F. Ismaiel , M. H. Abdelazim , I. Eldsoky , et al., “The Impact of COVID‐19 Outbreak on the Incidence of Acute Invasive Fungal Rhinosinusitis,” American Journal of Otolaryngology 42, no. 6 (2021): 103080. 34022619 10.1016/j.amjoto.2021.103080 PMC8120788

[28] S. Kulkarni , B. L. Jenner , and I. Wilkinson , “COVID‐19 and Hypertension,” Journal of the Renin‐Angiotensin‐Aldosterone System 21, no. 2 (2020): 1470320320927851. 32431227 10.1177/1470320320927851 PMC7249576

[29] B. Li , J. Yang , F. Zhao , et al., “Prevalence and Impact of Cardiovascular Metabolic Diseases on COVID‐19 in China,” Clinical Research in Cardiology 109 (2020): 531–538. 32161990 10.1007/s00392-020-01626-9 PMC7087935

[30] N. Sekar and K. T. Sundaresan , “The Co‐Infection of Mild COVID‐19 and Rhinocerebral Mucormycosis in a Patient Without Diabetes or Prior Steroid Use,” Cureus 14, no. 5 (2022), 10.7759/cureus.24986. PMC9189617 35719782

[31] O. A. Cornely , A. Alastruey‐Izquierdo , D. Arenz , et al., “Global Guideline for the Diagnosis and Management of Mucormycosis: An Initiative of the European Confederation of Medical Mycology in Cooperation With the Mycoses Study Group Education and Research Consortium,” Lancet Infectious Diseases 19, no. 12 (2019): e405–e421. 31699664 10.1016/S1473-3099(19)30312-3 PMC8559573

[32] R. Samson and M. Dharne , “COVID‐19 Associated Mucormycosis: Evolving Technologies for Early and Rapid Diagnosis,” 3 Biotech 12, no. 1 (2022): 6. 10.1007/s13205-021-03080-4 PMC8647065 34900512

[33] M. Hoenigl , D. Seidel , A. Carvalho , et al., “The Emergence of COVID‐19 Associated Mucormycosis: A Review of Cases From 18 Countries,” Lancet Microbe 3, no. 7 (2022): e543–e552. 35098179 10.1016/S2666-5247(21)00237-8 PMC8789240

[34] H. Guegan , X. Iriart , M.‐E. Bougnoux , A. Berry , F. Robert‐Gangneux , and J.‐P. Gangneux , “Evaluation of MucorGenius® Mucorales PCR Assay for the Diagnosis of Pulmonary Mucormycosis,” Journal of Infection 81, no. 2 (2020): 311–317. 32474046 10.1016/j.jinf.2020.05.051

[35] D. Gayathri and R. Soundarya , “An Emergence of Mucormycosis During the COVID‑19 Pandemic,” World Academy of Sciences Journal 6, no. 2 (2024): 13.

